# Validation of the Apple Watch for Estimating Moderate-to-Vigorous Physical Activity and Activity Energy Expenditure in School-Aged Children

**DOI:** 10.3390/s21196413

**Published:** 2021-09-25

**Authors:** Sunku Kwon, Youngwon Kim, Yang Bai, Ryan D. Burns, Timothy A. Brusseau, Wonwoo Byun

**Affiliations:** 1 Department of Health and Kinesiology, University of Utah, Salt Lake City, UT 84112, USA; sunku.kwon@utah.edu (S.K.); yang.bai@utah.edu (Y.B.); ryan.d.burns@utah.edu (R.D.B.); tim.brusseau@utah.edu (T.A.B.); 2 School of Public Health, Li Ka Shing Faculty of Medicine, The University of Hong Kong, Hong Kong 999077, China; youngwon.kim@hku.hk; 3 MRC Epidemiology Unit, School of Clinical Medicine, University of Cambridge, Cambridge CB2 0SL, UK

**Keywords:** moderate-to-vigorous physical activity, active energy expenditure, Apple Watch, children

## Abstract

The Apple Watch is one of the most popular wearable devices designed to monitor physical activity (PA). However, it is currently unknown whether the Apple Watch accurately estimates children’s free-living PA. Therefore, this study assessed the concurrent validity of the Apple Watch 3 in estimating moderate-to-vigorous physical activity (MVPA) time and active energy expenditure (AEE) for school-aged children under a simulated and a free-living condition. Twenty elementary school students (Girls: 45%, age: 9.7 ± 2.0 years) wore an Apple Watch 3 device on their wrist and performed prescribed free-living activities in a lab setting. A subgroup of participants (N = 5) wore the Apple Watch for seven consecutive days in order to assess the validity in free-living condition. The K5 indirect calorimetry (K5) and GT3X+ were used as the criterion measure under simulated free-living and free-living conditions, respectively. Mean absolute percent errors (MAPE) and Bland-Altman (BA) plots were conducted to assess the validity of the Apple Watch 3 compared to those from the criterion measures. Equivalence testing determined the statistical equivalence between the Apple Watch and K5 for MVPA time and AEE. The Apple Watch provided comparable estimates for MVPA time (mean bias: 0.3 min, *p* = 0.91, MAPE: 1%) and for AEE (mean bias: 3.8 kcal min, *p* = 0.75, MAPE: 4%) during the simulated free-living condition. The BA plots indicated no systematic bias for the agreement in MVPA and AEE estimates between the K5 and Apple Watch 3. However, the Apple Watch had a relatively large variability in estimating AEE in children. The Apple Watch was statistically equivalent to the K5 within ±17.7% and ±20.8% for MVPA time and AEE estimates, respectively. Our findings suggest that the Apple Watch 3 has the potential to be used as a PA assessment tool to estimate MVPA in school-aged children.

## 1. Introduction

Moderate-to-vigorous physical activity (MVPA) in children offers numerous health benefits, including the prevention of childhood obesity [1], improved bone strength [2], and improved cardio-respiratory endurance [3,4]. Given that physical activity (PA) monitoring plays an important role in promoting children’s MVPA [5], PA assessment, which focuses on estimating MVPA time and activity energy expenditure (AEE), can be useful for PA monitoring. Such quantification of activity intensities, time, and AEE in real-time has been made possible through the advancement of micro-technology and the deployment of various consumer-based wearable devices, such as activity trackers and smartwatches. Smartwatches, in particular, have recently been recognized as effective tools in monitoring PA patterns in both clinical and research settings due to wear convenience and real-time monitoring of steps, energy expenditure (EE), and intensity of activities [6,7,8].

Apple Watch^®^ (Apple Inc., Cupertino, CA, USA) is currently the most popular smartwatch available, possessing approximately 48% of the global market share in 2019 [9]. This device is equipped with accelerometers, gyroscopes, and altimeters that are used to estimate a user’s exercise time, total activity, moving distance, active EE, and steps. The Activity app on the Apple Watch offers a daily portrait of how much a user exercises, moves, and stands, and is paired with the accompanying Activity app on a smartphone to track PA patterns daily and in real-time. The apps are designed to allow users to set daily PA goals, earn activity awards, and compete with others in activity-based competitions. For example, Apple Watch users might earn a “Perfect Week (exercise)” when they meet their personal goals. These award features are designed to promote regular PA in users, and it indicated the Apple Watch is indeed a useful tool for increasing PA levels in adults [10]. These features, as well as the Apple Watch’s growing popularity and affordability, suggest that the Apple Watch has tremendous potential for use in PA epidemiologic research. Considering the recent increase in smart device use among children, the Apple Watch can also be an attractive tool for children to increase their PA engagement. Therefore, the data from the Apple Watch would allow researchers to better understand the way to promote PA engagement in children.

Previous studies for adults and youth have examined the validity of the Apple Watch’s EE estimation, and reported moderate to strong correlations (range: r = 0.71 to 0.88), and an acceptable measurement error (14.1 to 24.3%) against the indirect calorimetry [11,12,13]. In addition, the Apple Watch has been used as a measurement method for PA in a recent large-scale study, named “Apple Heart and Movement Study”, which examines any potential factors associated with heart health and PA in the cohort of Apple Watch users. In this particular study, participants were able to self-enroll using their Apple Watch, and researchers can remotely recruit participants and acquire participants’ data through the research app. 

The rapid rate of advancements in sensor technology promotes the use of device-based PA measurement, and the Apple Watch is one of the most popular and promising wearable devices for measuring PA in epidemiological research. Accordingly, it is essential to assess the validity of the Apple Watch in estimating children’s PA. Children typically have intermittent activity patterns and less accurate recall for their behavior, as a result of their less interest in continuous activity and a relatively short span of attention on a given task [14]. Assessing PA in children is challenging due to their intermittent activity patterns and limited ability to recall their behaviors [14,15,16]. Thus, accelerometry-based activity monitors have been recognized as a standard measure of habitual PA in children due to its objectivity, unobtrusiveness and accuracy. While the Apple Watch can be a useful PA measurement tool in children, the Apple Watch may not record some MVPA times in children due to their intermittent activity patterns. More specifically, Apple Watch captures MVPA time in minutes, and it may not recognize the span as MVPA time if the total amount of activity within 1 min is less than the set volume as MVPA, due to intermittent activity patterns during continuous activity. However, there has been sparse research on whether the Apple Watch accurately estimates the time engaged in MVPA and activity EE (AEE) in school-aged children. 

Since 2021, the Apple Watch 3 has become affordable, and has the same features on the monitoring of fitness and PA compared to the Apple Watch 6, which is the newest model. Moreover, the ability to manage multiple Apple Watches from a single iPhone through a family account can support researchers to use of Apple Watch 3 in the research aimed at measuring and promoting children’s PA. As the Apple Watch 3 can be utilized as a measurement tool in a large-scale cohort PA study, it is also essential to determine how the Apple Watch performs in children. Therefore, the purpose of this study was to examine the concurrent validity of the Apple Watch 3 for estimating MVPA time and AEE in elementary school-aged children under a simulated activity setting and free-living condition.

## 2. Materials and Methods

### 2.1. Participants

A convenience sample of 20 elementary school-aged children (Girls: 50%, Age: 9.7 ± 1.9 years, BMI percentile: 36.9 ± 23.1%) was recruited via email, flyers, and word-of-mouth. Children who were physically disabled or otherwise unable to participate in PA were excluded from this study. The study protocol was approved by the Institutional Review Board (IRB) of the University of Utah (IRB approval number: 00108150). Participants and their parents provided signed assent and informed consent prior to participation in this study. 

### 2.2. Instruments

#### 2.2.1. Apple Watch

The Apple Watch 3 is a wrist-worn smart device (weight range: 26.7 to 52.8 g) that includes a retina OLED display (size: 38 or 42 mm), tri-axial accelerometer (up to 16 g-forces), tri-axial gyroscope, barometric altimeter, optical heart sensor, and global positioning system. This device is water-resistant (up to 50 m) and has up to 18 h of battery life. The Apple Watch 3 is advertised to estimate exercise minutes, active and resting calories, steps, distance, and standing hours by accelerometer, gyroscope, and barometric altimeter in real-time and per day. Moreover, this device can monitor heart rates in real-time using the optical heart sensor. The activity app built in the Apple Watch 3 offers individuals the ability to track PA and set daily PA goals. Further, the app shows total calories by the sum of active and resting calories. The Apple Watch 3 was placed on the dominant wrist of the participants following the manufacturer’s recommendations and connected to an accompanied iPhone throughout the study.

#### 2.2.2. Indirect Calorimetry

Cosmed K5 (K5; COSMED, Rome, Italy) served as a criterion method for measuring MVPA and AEE during the lab session. The K5 is a valid and reliable portable indirect metabolic system that can accurately measure respiratory minute volume (VE), oxygen uptake (VO_2_), and carbon dioxide production (VCO_2_) using a breath-by-breath method [17,18,19]. Moreover, previous studies in adults and children used the portable indirect calorimeter as a criterion measurement to examine the validity of various wearable activity monitors in estimating PA intensities [20,21]. The main unit of the K5 (174 × 111 × 64 mm and 900 g, including battery and Oxygen (O_2_) sensor) is placed on the participant’s upper back using an adjustable harness. The main unit of the K5 communicates with a computer by Bluetooth to record and store the measured data. Before data collection, the K5 was calibrated following the manufacturer’s recommendation. The current study assessed MVPA time and AEE of participants using the measured VO2 values from the K5 [19]. 

#### 2.2.3. ActiGraph GT3X+ 

The ActiGraph GT3X+ (GT3X+; ActiGraph, Pensacola, FL, USA) is a small and light (4.6 × 3.3 × 1.5 cm; 19 g) research-grade accelerometer that can be worn on the wrist or at the waist using a manufacturer-provided wrist-strap or waist strap. This device records raw accelerations in three axes with a dynamic range ± 6 g at a user-specified sampling frequency (30–100 Hz). Also, the GT3X+ can estimate activity and sedentary bout, PA intensity, and steps taken at a user-selected epoch length (1–60 s) [22]. The device has been validated for its accuracy in estimating PA compared to the measures from indirect calorimetry in adults and children [23,24,25], and has been widely used as a criterion measure for evaluating the validity of consumer-based activity monitors in estimating PA under a free-living condition [26,27]. In the current study, the GT3X+ was used as a criterion measure of MVPA under the free-living condition.

### 2.3. Procedures

Participants, accompanied by a parent, visited the Physical Activity Research Laboratory at the University of Utah for their lab session. Prior to the lab session, participants completed the informed consent and a demographic questionnaire. Trained research staff measured participants’ height (cm), weight (kg), and waist circumference (cm) using a wall stadiometer (ShorrBoard^®^, Olney, MD, USA), an electric body scale (Seca 869, Hamburg, Germany), and a tape measure (Baseline^®^ Evaluation Instruments, White Plains, NY, USA), respectively. Body mass index (BMI) was calculated using the measured height and weight. The measured anthropometric characteristics were entered in the Apple Watch 3’s Activity app, and the K5 software to initialize the Apple Watch 3 and the indirect calorimetry for each individual’s testing. 

The Apple Watch 3 was placed on a participant’s dominant wrist before the participant was fitted with K5 to measure breath-by-breath oxygen uptake during the lab session. MVPA (i.e., exercise minutes) and AEE (i.e., active calories) values of the Apple Watch 3 were recorded at the beginning of the activity protocol. Following these preparations, each participant performed a 50-min activity protocol, which included resting, simulated free-living activities, and 1-min transition periods. Initially, participants were taken a rest in an inclined position for three minutes. The resting was followed by a total of 14 activities in a gymnasium. The activities were selected to simulate typical activities for children in free-living, according to the youth PA compendium [28]. Participants selected their preferred activities to perform across ranges of activity intensities. Research staff tracked the time of each activity, and provided verbal cues to the participants to transition to the next activity.

To evaluate the accuracy of the Apple Watch 3 in estimating time spent in MVPA under the free-living condition in a field setting, five participants were randomly selected as a subsample group following the lab session. The selected participants were asked to wear the GT3X+ and Apple Watch 3 on their non-dominant and dominant wrists, respectively, and went about their daily life in free-living conditions for at least 7 consecutive days. Prior to deployment, the GT3X+ was initialized with a sampling rate of 30 Hz using the ActiLife software (ActiGraph, Pensacola, FL, USA). Participants were required to take off both devices during any aquatic activities and sleep time. Parents recorded their children’s non-wear and sleep time on the sleep-activity log sheet. 

### 2.4. Data Processing

Upon completing the lab session, the estimated MVPA and AEE from the Apple Watch 3 were immediately recorded. The metabolic equivalence of tasks (MET) were calculated using the measured VO2 (mL/min) from the K5 and each participant’s body weight (kg). The average value of the metabolic rates during the resting period was used as 1-MET to classify the children’s PA intensity. The calculated MET were classified with different activity levels (≤1.5 MET = SED, 1.6–2.9 MET = LPA, 3.0–5.9 MET = Moderate PA, and ≥6.0 MET = Vigorous PA). For comparison to the Apple Watch 3’s AEE estimates, the measured net AEE of the testing day was calculated. First, resting, sedentary activity, and transition periods were removed to leave only active minutes. The measured VO_2_ data (mL/min) were multiplied by 1000 to obtain VO_2_ in L/min, and then multiplied by 4.867 kcals/L to obtain kcals/min. These calculated kcals/min values were summed to obtain the total AEE of the testing day. In addition, the basal metabolic rate (kcal/day) was predicted for each participant using the Schofield equations [29]. The predicted basal metabolic rate was divided by 1440 min to calculate kcals/min values, then multiplied by the total minutes of active time. The calculated total basal metabolic rate was subtracted from the calculated total AEE to obtain the net AEE for the testing day. The processed K5 data were aggregated to the daily average, then merged and aligned with the Apple Watch 3 data for statistical analyses. 

For the subsample group, daily MVPA estimates of the Apple Watch 3 were obtained from the iPhone’s Activity app. Data from the GT3X+, under the free-living condition, were downloaded in a raw acceleration data format and converted into “.csv” files using ActiLife software. The raw acceleration data were processed in R software (http://cran.r-project.org; accessed on 22 November 2020) using the GGIR package (version 1.10–10) [30]. The GGIR package calibrated the raw tri-axial accelerations derived by the GT3X+ and converted it to the Euclidean norm minus one (ENMO;$\;\sqrt{x^{2}+y^{2}+z^{2}}-1\;g$), which indicates the value of gravity with negative values rounded to zero [31]. The ENMO values were classified as different activity levels per one-minute using the intensity thresholds for ENMO derived by Hildebrand et al. [25,32]. Moreover, periods of non-wear and sleep were identified and excluded using Choi’s algorithm [33]. The self-reported activity/sleep logs from each participant were also excluded. The processed GT3X+ data were aggregated to the daily average, then merged and aligned with the daily MVPA estimates of the Apple Watch 3 for statistical analyses. 

### 2.5. Statistical Analyses 

Descriptive analyses were conducted to summarize the demographic and anthropometric characteristics of the participants. 

Mean absolute percent errors (MAPEs) were calculated to evaluate the measurement error of the Apple Watch 3 in the estimation of MVPA time and/or AEE compared to those from the criterion measures (i.e., |(criterion − estimation)/criterion| × 100). 

Bland-Altman (BA) plots were used to evaluate the agreement and systematic biases in estimating time spent in MVPA and AEE between the Apple Watch 3 and the K5. The mean bias (i.e., criterion − estimation) was computed to provide the overall overestimation or underestimation of the Apple Watch 3 for MVAP time, as well as the AEE compared to criterion measure. The significance of systematic bias was determined by whether the 95% confidence interval of the mean bias included the line of equality (i.e., criterion − estimation = 0). Moreover, the limits of agreement were calculated as mean bias ±1.96 standard deviation for evaluating the individual-level agreement. 

Finally, an equivalence test was conducted to determine the equivalence at the group level between the K5 and Apple Watch 3 in estimating MVPA and AEE. The 90% confidence interval (CI) of the estimates from the Apple Watch 3 was compared with the equivalence zone (EZ) from the K5 measures. Given no evidence presently exists of a universally accepted EZ range, the current study established the minimal EZs of the K5 measures that include the 90% CIs of the Apple Watch 3 estimates. Data were analyzed using Stata 14.2 software (StataCorp LLC, College Station, TX, USA) and SAS 9.4 software (SAS Institute, Cary, NC, USA), and statistical significance was determined at *p* < 0.05.

## 3. Results

Participant characteristics are summarized in Table 1. There were no significant differences in age, height, weight, BMI percentile, and waist circumference between boys and girls. Table 2 presented the mean differences, and MAPE values of MVPA and AEE estimates, in the K5 and Apple Watch 3. As shown in Table 2, the results of the paired t-test revealed no significant differences in MVPA estimates between the K5 and Apple Watch 3. The MAPE in Apple Watch 3 was 1% for MVPA estimate compared to the K5 measures. For the AEE, the Apple Watch 3 underestimated AEE by 4%, but the mean difference was not statistically significant (mean difference: 3.8 kcal, *p* = 0.75). With respect to AEE, the Apple Watch 3 also had a minimal measurement error (4%) relative to the measured AEE from the K5. 

**Figure 1:**
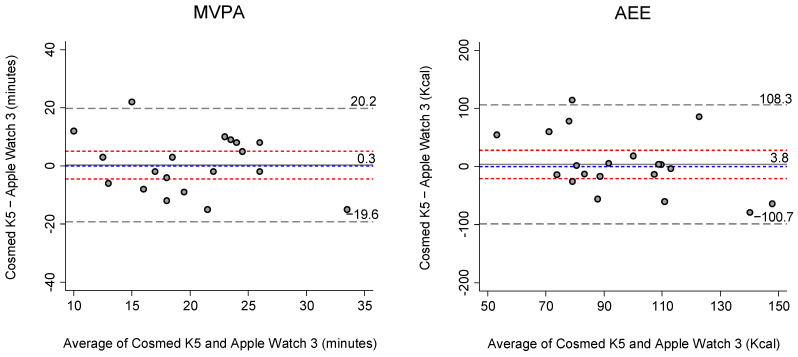
Bland-Altman Plots for comparing Moderate-to-Vigorous Physical Activity (MVPA) and Active Energy Expenditure (AEE) estimates between the K5 and Apple Watch 3. Blue short-dashed lines show the line of equality. Solid lines and red short-dashed lines indicate mean bias and 95% confidence interval of the mean bias, respectively. Dashed lines show 95% limits of agreement (±1.96 standard deviation).

Bland-Altman plots (Figure 1) illustrated the agreement between the K5 and Apple Watch 3 for MVPA and AEE estimates by displaying the mean difference and level of agreement. The BA plots showed that there was no apparent bias for the agreement in MVPA estimates between the K5 and Apple Watch 3. For the AEE estimates, however, the Apple Watch 3 had a wide 95% limit of agreement (−100.7 to 108.3 kcal) compared to the K5 measures. 

**Figure 2:**
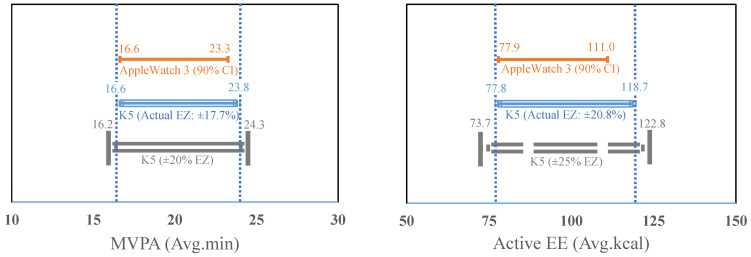
Equivalence Testing for MVPA and AEE estimates between the K5 and Apple Watch 3. CI: Confidence interval; EZ: Equivalence zone; MVPA: Moderate-to-vigorous physical activity; EE: Energy Expenditure; vertical dashed lines show the actual bounds within which the Apple Watch 3 is statistically equivalent to the K5 for MVPA and Active EE estimates.

Figure 2 shows the results of the equivalence tests. The 90% confidence interval (CI) of the MVPA estimates from the Apple Watch 3 (90% CI = 16.63 to 23.36 min) fell within ±17.7% EZ (16.63 to 23.89 min) of the measured MVPA from the K5. Furthermore, the EZ of AEE measured by the K5 was established ±20.8% (77.8 to 118.67 kcal), which included the 90% CI of the AEE estimate (77.87 to 111.03 kcal) from the Apple Watch 3.

In a subsample group, we found that the Apple Watch 3 had a relatively large mean bias (Mean difference: −25.3 ± 4.5) and high MAPE (47.5%) in estimating time spent in MVPA compared to the estimates from the GT3X+ in the free-living condition (Table 3).

## 4. Discussion

To our knowledge, this is the first study to investigate whether the Apple Watch 3 accurately estimates time spent in MVPA and AEE compared to the established criterion measures for elementary school-aged children. The results from this study indicated that the Apple Watch 3 can provide comparable estimates for MVPA time against the indirect calorimetry in elementary school-aged children. We also observed that the Apple Watch 3 had no apparent systematic bias in estimating children’s AEE relative to the indirect calorimetry, but the variability of the AEE estimates was relatively large. An advantage of the Apple Watch over the research-grade accelerometers is the ability to provide the incorporated PA data without device retrieving and data processing via remote monitoring. Accordingly, utilizing the data from the Apple Watch facilitates large-scale surveillance, which aims to promote PA in school-aged children. Given the practical applications of the Apple Watch 3 in future PA research, our findings provide important implications for researchers. The Apple Watch 3 can be a considerable device for monitoring children’s MVPA.

### 4.1. Accuracy of the Apple Watch 3 in Estimating MVPA

A notable finding is that the Apple Watch 3 can accurately estimate children’s MVPA time. More specifically, the Apple Watch 3 showed a small mean bias (0.3 min) and measurement error (MAPE: 1%) in estimating MVPA time compared to the indirect calorimetry, and the systematic bias was not significant. These findings indicate that Apple Watch 3 has comparable accuracy to the measures from the indirect calorimetry both at the group and individual levels in estimating MVPA in children. Although habitual time spent in MVPA is important to prevent childhood obesity, the MVPA time in children tends to gradually decline from age 8, and the declines are most pronounced at age 9 for both boys and girls [34]. Given that accurate PA monitoring is a key component for the promotion of children’s MVPA level [5], accelerometry-based activity monitoring is a reasonable method to objectively assess MVPA time for school-aged children who have a limited ability to recall their habitual activities [35]. More specifically, wearable activity monitors, embedded in a triaxial accelerometer, may provide valid estimates for children’s MVPA, which commonly includes intermittent activity patterns by incorporating accelerations derived from three directions (i.e., vertical, mediolateral, and anteroposterior). Furthermore, an addition to heart rate monitoring may make up for the biomechanical errors associated with accelerometry, thus enhance the accuracy of activity estimates in children during exercise [36,37]. As the Apple Watch 3 includes a heart rate monitor as well as a tri-axial accelerometer, it is speculated that the Apple Watch 3 might determine the user’s exercise minutes using a set of algorithms that integrate both the accelerometer and heart rate data to improve the accuracy of MVPA time measurement. Accordingly, the Apple Watch 3 can allow researches to evaluate the time spent in MVPA during the diverse activities in which children partake. Considering the observed accuracy of the Apple Watch in estimating MVPA time, the Apple Watch 3 would be a viable measurement device in future studies, which aim to increase MVPA levels in school-aged children. 

### 4.2. Validity of the Apple Watch 3 in Estimating AEE

Another important finding is that the present study showed a contrary result to a previous finding that the Apple Watch significantly underestimated AEE in youth [21]. The study by LaMunion et al. reported that the Apple Watch significantly underestimated AEE by 45% (mean difference: −121.8 kcal) and had more than 40% measurement error for AEE estimates compared to indirect calorimetry in youth [21]. However, the present study showed that the Apple Watch 3 similarly estimated AEE (mean difference: −3.8 kcal) with a relatively low measurement error (4%) compared to the indirect calorimetry in children. One possible explanation for the difference in AEE estimation is that the Apple Watch might be sensitive to body movements in estimating AEE [11]. While the current study evaluated the AEE during a simulated free-living activity protocol, LaMunion’s study included stationary cycling, which leads to less arm movement during physical activity [21]. Also, LaMunion’s study included 39 adolescents between 13 and 18 years old [21]. Adolescents may have relatively less body movement than children when consuming the same calories [38]. It should also be noted that LaMunion’s study used the previous generation of the Apple Watch used in the current study. However, given that the proprietary algorithm is confidential, it is unknown if or how the manufacturer updated the energy expenditure prediction algorithm when new models were released. 

It is noteworthy that the Apple Watch 3 has a relatively large inter-individual variability for EE estimation in children. The present study defined the expected limits of maximum acceptable bias (i.e., limits of agreement) of the Apple Watch in estimating children’s EE through the Bland-Altman plots [39]. The result from the Bland–Altman plots across all activities revealed that there was no significant systematic bias with a relatively small mean bias (3.8 kcal) because the line of equality was within the confidence interval of the mean bias (Figure 1). However, the limit of agreement for the Apple Watch’s EE estimation was relatively wider (−100.2 to 108 kcal) than the limits of agreement from previous studies, which include adults (−54.5 to 124.5 kcal) and adolescents (98.1 to 251.2 kcal) [12,21]. For this reason, it is premature to recommend the widespread use of the Apple Watch 3 for the assessment of children’s EE in free-living environments. The ability to assess energy expenditure in children is a clinically important component to children’s PA research for non-communicable diseases, including malnutrition, obesity, and diabetes. In this regard, the findings from the current study are critical when researchers consider using AEE data from the Apple Watch to facilitate PA in a manner appropriate prevention of childhood obesity. However, future research is still warranted to further explore the practical application of the Apple Watch 3 in estimating children’s AEE under free-living conditions. 

### 4.3. Practical Applications and Considerations 

A novel aspect of this study was the assessment of the relative equivalency of the Apple Watch for MVPA time and AEE estimations against the criterion measures. Although equivalence testing is a widely accepted analytic method to rigorously examine the agreement to the criterion measures in a dichotomous manner at a group level, the present study has not attempted to assess the agreement in the traditional way, as there is no universally acceptable equivalence zone range for the Apple Watch. Instead, the equivalence test was used to identify the actual equivalence zone where the 90% confidence intervals of the estimates from the Apple Watch 3 completely fell within the mean values from the K5 indirect calorimetry. Accordingly, the relative equivalency approach would make it possible to determine the acceptable measurement error of the Apple Watch in estimating MVPA time and AEE. With this analytic approach, we were able to determine the actual equivalence zone for MVPA time and AEE estimates as ±17.7% (16.63 to 23.89 min) and ±20.8% (77.8 to 118.67 kcal), respectively. We could also identify that the actual equivalence zones of indirect calorimetry to the Apple Watch’s AEE estimates from previous studies presented ±18% in adults and ±20% in youth [12,21]. It is noteworthy that the acceptable measurement error of the Apple Watch on AEE estimation would be less than 20.8% for school-aged children, which is higher than that for adults and youth. Therefore, the present study reveals the range of acceptable measurement errors of the Apple Watch in estimating MVPA time and AEE for children at the group level, and these findings are practically significant to researchers given the Apple Watch’s real-world application in school-aged children.

In the current study, we examined the validity of the Apple Watch 3 in estimating MVPA under a free-living condition. Compared to the GT3X+, on average, the estimated daily PA time from the Apple Watch 3 (648 min/day) was higher than the estimate of total PA from the GT3X+ (604 min/day). Therefore, researchers need to be aware that the Apple Watch 3 may slightly overestimate the amount of total PA in free-living conditions, compared with the GT3X+. 

There are several considerations to be considered when the Apple Watch is used as a measurement tool for PA research in children. First, as observed in previous studies, the Apple Watch may over- or under-estimate AEE and total EE [11,21,40,41,42]. In addition, current and previous studies indicated that Apple Watch’s error in estimating AEE varies widely among children. Moreover, it is likely that the Apple Watch could underestimate the AEE due to the intermittent activities of children. Thus, further investigations are warranted to assess the validity and reliability in estimating AEE under free-living conditions in children. Second, appropriate PA estimates need to be selected for investigating or promoting PA using the Apple Watch in children. The Apple Watch tracks various PA metrics to encourage healthy behaviors. The PA metrics include standing minutes per hour, exercise minutes (i.e., MVPA), and amounts of active calories. Of these, MVPA time tracking is an essential factor in evaluating whether the level of PA is adequate in children. Further, the use of wearable activity monitors is effective at increasing inactive children’s PA level by incorporating self-monitoring and goal setting for MVPA [8]. Lastly, using Apple Watch, researchers can collect multiple physiological (i.e., heart rate, blood pressure) and behavioral (i.e., PA) profiles in children with minimal burden. The usability of the device could be greater with the Apple Watch than research-based activity monitors because the Apple Watch provides more age-appropriate and interactive features through various compatible apps. In addition, researchers can encrypt the data collected from the research app, and securely store the collected data in a specific cloud system that technically safeguards the requirement of the Health Insurance Portability and Accountability Act. In support, a recent large-scale clinical trial called “Apple Heart and Movement Study” explores potential factors associated with heart health and PA over time using data from the Apple Watch. Given that Apple Watch users self-enroll in this trial, the project is able to recruit approximately 500,000 participants and remotely acquire participants’ data through the research app. In light of the validity and usability, using Apple Watch in research would help find new interventions that facilitate children to replace their sedentary behavior with an active lifestyle.

### 4.4. Strengths and Limitations

There are several strengths in this study. The main advantage of this study was the evaluation of accuracy in estimating both MVPA time and AEE in the Apple Watch 3 compared to indirect calorimetry. The indirect calorimetry system is regarded as a gold standard criterion measure to assess time engaged in sedentary behavior and PA, and AEE estimates during activities [11,12,20,21]. Furthermore, the investigation of both MVPA time and AEE estimates could provide better evidence if the Apple Watch 3 comparably estimates net AEE throughout the PA, which results in EE. The current study examined the validity of MVPA time and AEE estimates on the most popular smartwatch. The Apple Watch can be utilized in mobile healthcare systems through continuous updates of operating systems and health-related apps. Moreover, the Apple Watch can be connected to a specific app to deliver health intervention or access PA data monitored in the watch by other smart devices, such as iPhone. Thus, our findings might provide beneficial information to software developers and manufacturers, in order to update the software and hardware of the Apple Watch.

The current study also has several limitations. First, this study included a relatively small number of participants, which may limit the generalizability of the findings from this study; however, we assessed the validity of the Apple Watch 3 using rigorous statistical analytic methods (i.e., MAPEs, BA plots, equivalent tests) [43]. Therefore, the internal validity of this study is not threatened by the sample size. Lastly, given that the criterion measure under the free-living condition (GT3X+) is not completely waterproof, albeit water resistant (1 meter, 30 min), this study could not include any aquatic activities that may be popular in children. 

## 5. Conclusions

Compared to the measures from indirect calorimetry, the Apple Watch 3 comparably estimated time the spent in MVPA and AEE under the simulated free-living condition in elementary school students. The findings indicate that the Apple Watch 3 can be used in estimating MVPA time in PA research and health promotion programs. However, it is still unclear whether the Apple Watch 3 can be a valid wearable device for measuring AEE in children. Furthermore, the current study could not reveal whether the Apple Watch 3 accurately estimates MVPA time in children under true free-living conditions. The Apple Watch is a feasible device to measure PA information in research that promotes MVPA and an active lifestyle in children. Further research is warranted to evaluate the validity and reliability of the Apple Watch 3 in estimating children’s MVPA time and AEE under free-living conditions.

## Figures and Tables

**Table 1:** Descriptive characteristics of participants, mean ± standard deviation.

Characteristic	All (N = 20)	Boys (N = 11)	Girls (N = 9)	*p*-Value *
Age (years)	9.7 ± 2.0	9.7 ± 1.8	9.7 ± 2.2	0.95
Height (cm)	138.3 ± 13.4	138.9 ± 12.7	137.6 ± 14.9	0.84
Weight (kg)	31.8 ± 11.1	32.0 ± 9.4	31.5 ± 13.5	0.92
BMI (kg/m^2^)	16.3 ± 3.2	16.3 ± 2.4	16.2 ± 4.1	0.95
BMI percentile (%)	36.9 ± 29.1	35.0 ± 28.8	39.1± 31.1	0.76
Waist Circumference (cm)	60.9 ± 9.3	61.4 ± 9.5	60.2 ± 9.61	0.78

* *p*-value for gender difference.

**Table 2:** Estimated mean (SD), mean difference (SE), mean absolute percent error between indirect calorimetry and the Apple Watch 3 under the simulated free-living condition.

PA Metrics	Cosmed K5 (SD)	Apple Watch 3 (SD)	Mean diff. (SE)	MAPE (%)
MVPA	20.2 min (6.7)	19.9 min (8.3)	−0.3 min (2.3)	1%
AEE	98.2 kcal (25.6)	94.5 kcal (42.9)	3.8 kcal (11.7)	4%

MVPA: moderate-to-vigorous physical activity; AEE: active energy expenditure; MAPE: mean absolute percent error.

**Table 3:** Estimated mean (SD), mean difference (SE), mean absolute percent error between the GT3X+ and Apple Watch 3 under the free-living condition.

PA Metrics	Cosmed K5 (SD)	Apple Watch 3 (SD)	Mean diff. (SE)	MAPE (%)
MVPA	53.3 min (13.0)	78.6 min (21.3)	25.3 min (4.5)	47.5%

MVPA: moderate-to-vigorous physical activity; MAPE: mean absolute percent error.

## Data Availability

The datasets of the current study are available from the authors on reasonable request.
